# Endovascular retreatment of a splenic artery aneurysm refilled by collateral branches of the left gastric artery: a case report

**DOI:** 10.1186/1752-1947-8-436

**Published:** 2014-12-17

**Authors:** Anna Maria Ierardi, Mario Petrillo, Alessandro Bacuzzi, Chiara Floridi, Gianlorenzo Dionigi, Gabriele Piffaretti, Gianpaolo Carrafiello

**Affiliations:** 28 Department of Radiology, University of Insubria, Ospedale di Circolo e Fondazione Macchi, Viale Borri 57, 21100 Varese, VA Italy; 29 Radiology Department, Second University of Naples, Piazza Miraglia 8, 84010 Naples, NA Italy; 30 Anaesthesia and Palliative Care, University of Insubria, Ospedale di Circolo e Fondazione Macchi, Viale Borri 57, 21100 Varese, VA Italy; 31 Department of Surgical Sciences, University of Insubria, Ospedale di Circolo e Fondazione Macchi, Viale Borri 57, 21100 Varese, VA Italy; 32 Vascular Surgery Department, University of Insubria, Ospedale di Circolo e Fondazione Macchi, Viale Borri 57, 21100 Varese, VA Italy

**Keywords:** Endovascular treatment, Left gastric artery supplying, Splenic aneurysm

## Abstract

**Introduction:**

A rare case of a splenic artery aneurysm refilled by a hypertrophic branch originating from the left gastric artery retreated with an endovascular approach is reported. To the best of our knowledge, this is the first such case reported in the literature.

**Case presentation:**

A hilum splenic artery aneurysm of a 43-year-old Caucasian woman was treated with endovascular ligature. Contrast-enhanced computed tomography performed after 1 month revealed reperfusion of the aneurysm and a new angiogram demonstrated a hypertrophic vessel from her left gastric artery supplying the sac of the aneurysm. It was catheterized by splenic hilum branches and it was embolized with coil and glue. Contrast-enhanced computed tomography performed after 3 months confirmed complete exclusion of the sac of the aneurysm.

**Conclusions:**

Our patient represents the first rare case of a splenic artery aneurysm refilled from a branch of her left gastric artery not visible at first at angiography or at contrast-enhanced computed tomography performed after 1 month; it was revealed at the second angiography and it was definitively embolized. These eventualities and possibilities of treatment, although rare, should be kept in mind for each patient with similar presentation.

## Introduction

Splenic artery aneurysm (SAA) is the most common abdominal splanchnic artery aneurysm, accounting for approximately 60% of all such aneurysms [1].

The etiology of the disease is unknown, but has been connected to several other conditions, including atherosclerosis, hypertension, portal hypertension, cirrhosis, liver transplantation, and pregnancy [2].

These aneurysms are seen almost four times more often in women than in men, but are more likely to rupture in men [2, 3]. The reported risk of rupture ranges from 3% to 10%, and, when rupture occurs, mortality is seen in 20% to 100% of patients [4]. The general consensus is that SAAs greater than 2cm in size should be treated to avoid the complications of rupture [3, 4]. For some time, transcatheter embolization has been a safe and effective treatment option for noncomplicated SAAs [4, 5]. Although no randomized controlled trial exists in evaluating the efficacy of this procedure, there are several case reports and small or generalized retrospective reviews [4–6].

We describe a case of an aneurysm of the distal tract splenic artery, treated twice by coils embolization for the presence of a rare reperfusion after first treatment by an afferent of the left gastric artery. The exclusion of the aneurysm was obtained and the patency of the splenic artery was preserved without technical complications. A hypertrophic vessel afferent from the left gastric artery as cause of a SAA reperfusion may be considered a very rare event.

## Case presentation

**Figure 1:**
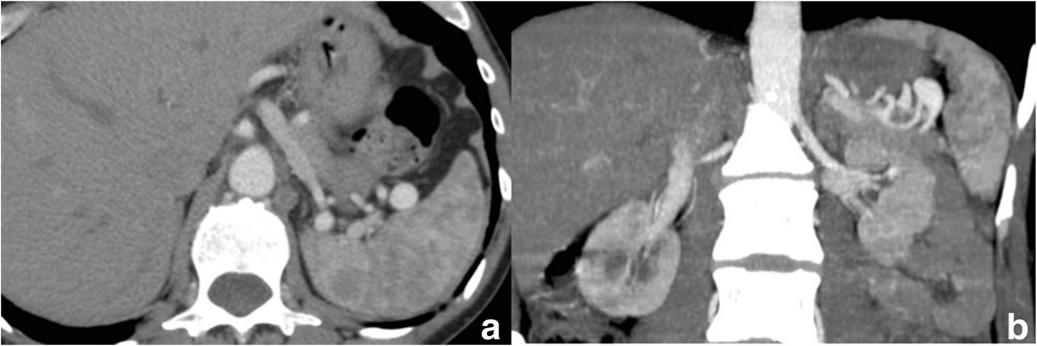
**(a,b): Computed tomography scan shows splenic artery aneurysm (a); coronal view better reveals afferent and efferent vessels of the aneurismal sac (b).**

**Figure 2:**
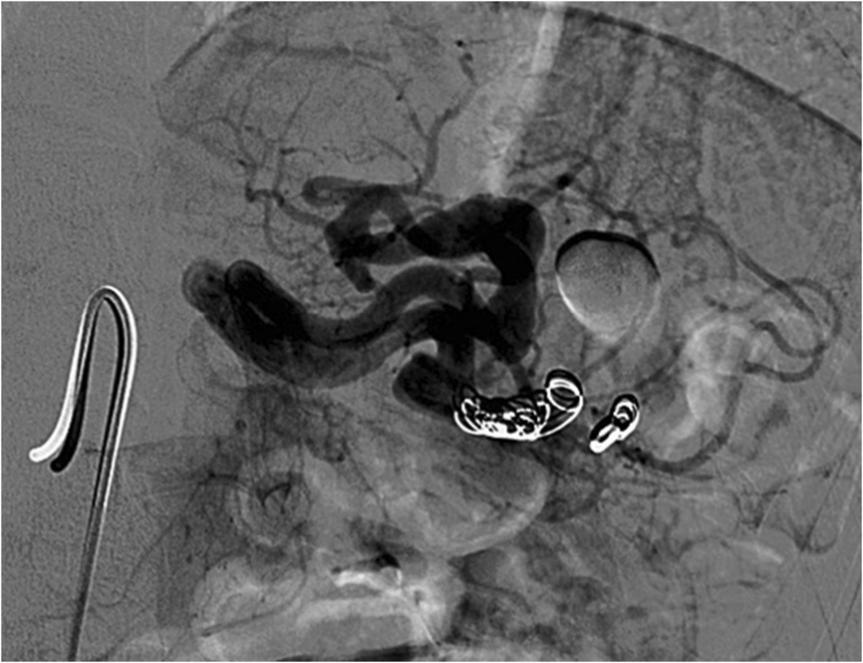
**Angiography after endovascular ligature showed exclusion of the aneurysmal sac.**

A 43-year-old Caucasian woman was referred to our hospital with a complaint of epigastric discomfort. Abdominal ultrasonography and contrast-enhanced computed tomography (CT) showed a sole SAA with a maximum diameter of 20mm (Figure 1a, 1b). No other concomitant disease, like cirrhosis, portal hypertension, collagen vascular disease or other abnormalities, was observed. Multiplanar and three-dimensional imaging was performed for subsequent planning of treatment. Endovascular treatment with endovascular ligature was proposed. A right common femoral artery approach was performed under local anesthesia, a 5-French sheath was placed, and diagnostic splenic angiograms were initially obtained with selective catheterization of the celiac trunk, which confirmed an aneurysm of the distal tract of her splenic artery. Considering the tortuosity of the splenic artery a microcatheter (Progreat®, Terumo, Tokyo, Japan) was placed into her splenic artery distal to the aneurysm to achieve distal embolization. A detachable coil (Interlock™, Boston Scientific, Natick, Massachusetts, USA) 6mm×10cm was deployed. After microcatheter retraction proximal to the aneurysm, a second detachable coil (Interlock™, Boston Scientific, Natick, Massachusetts, USA) 5mm×15cm was deployed. Angiography after endovascular ligature showed exclusion of the aneurysmal sac (Figure 2). Color Doppler ultrasound (CDU) and contrast-enhanced ultrasound (CEUS) performed the day after the procedure, showed devascularization of the SAA. Examinations showed ischemic damage of her spleen, but most of the splenic parenchymal vascularization was present.

**Figure 3:**
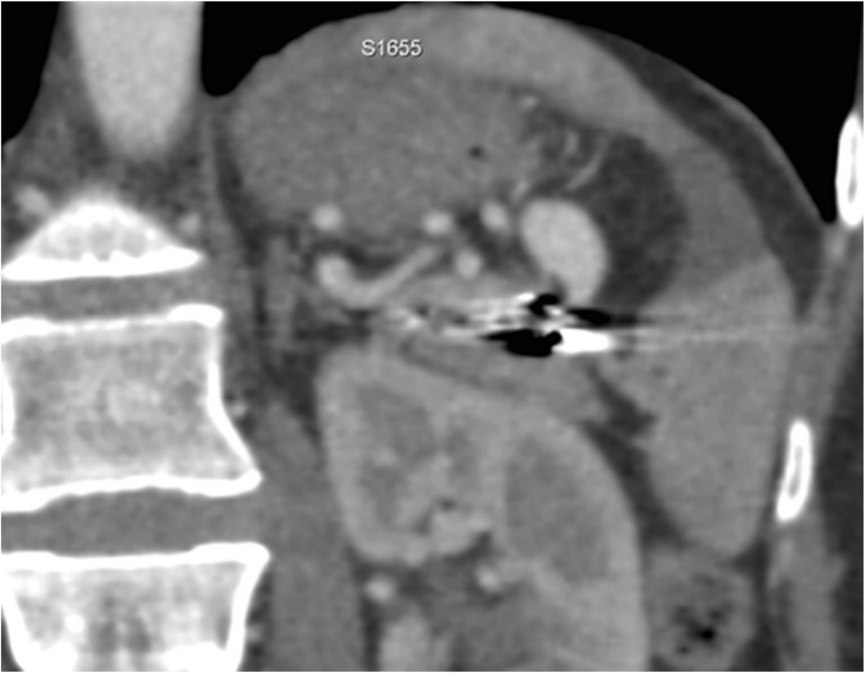
**Computed tomography scan (coronal view) revealed the reperfusion of the aneurysmal sac.**

**Figure 4:**
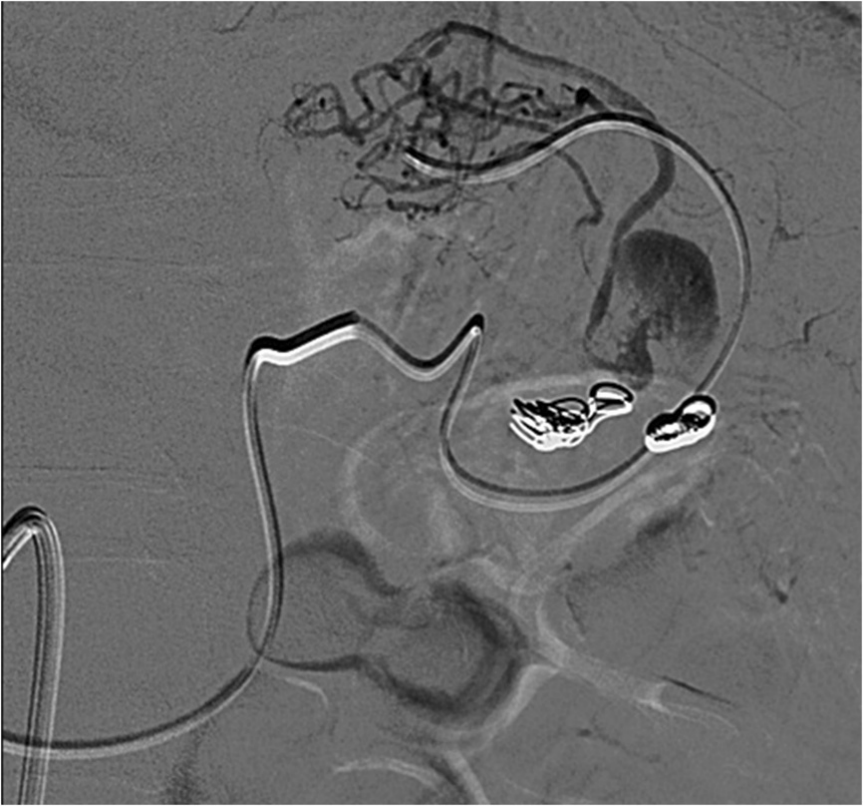
**Angiogram reveals an afferent vessel to the sac of the aneurysm originating from the left gastric artery.**

**Figure 5:**
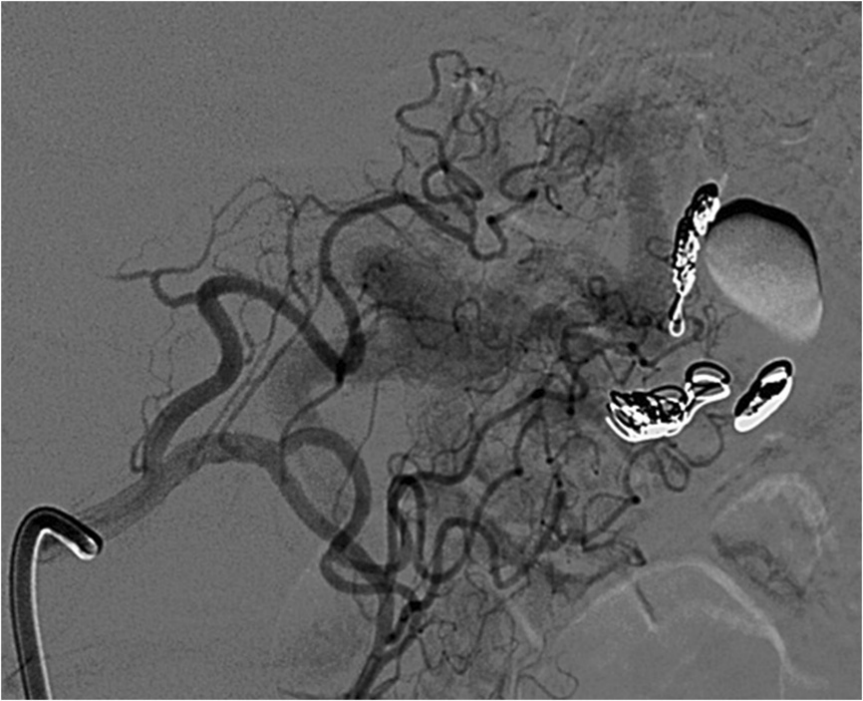
**Angiogram performed at the end of the procedure showed exclusion of the aneurysmal sac.**

**Figure 6:**
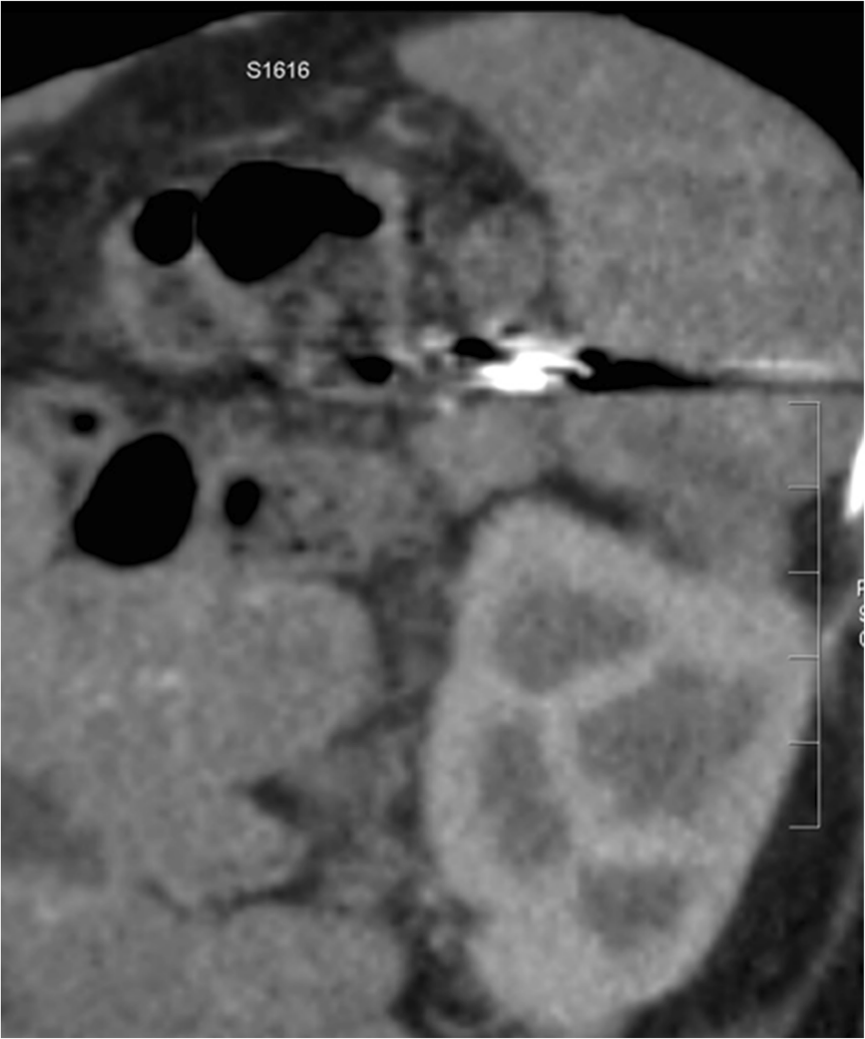
**Computed tomography scan (coronal view) performed 3 months after the second procedure confirmed the complete exclusion of the splenic artery aneurysm.**

Although symptoms indicative of mild post-embolization syndrome, such as transient fever and pain, were observed in our patient, administration of antibiotics and analgesic-antipyretic drugs was effective and no refractory symptoms were noted. Two days after the procedure she was discharged. The first imaging examination of follow up consists of a contrast-enhanced CT after 1 month. CT images and three-dimensional reconstructions revealed a persistent perfusion of the sac of the aneurysm; on the basis of the images the cause of the reperfusion was not clear, we hypothesized an incomplete embolization of the coils previously deployed (Figure 3). A second hospitalization was organized and a second angiography was performed. A selective angiogram of the celiac trunk revealed an afferent vessel to the sac of the aneurysm originating from her left gastric artery and confirmed absence of reperfusion by splenic vessels previously embolized (Figure 4). A superselective catheterization of that vessel was attempted but the onset of the vasospasm prevented it. The day after, a second attempt was performed, and the vessel was catheterized through splenic hilum vessels anastomosed with branches of her left gastric artery. We used a 2.7 French microcatheter (Progreat®, Terumo, Tokyo, Japan) and a second smaller microcatheter 2.1 French (Echelon™-10, EV3, Paris, France) was introduced within the first one. The vessel afferent the aneurysmal sac was visualized and selectively catheterized. It was embolized with 2 microcoils 4mmx10cm (VortX®; Boston Scientific, Natick, Massachusetts, USA) and 1cc of glue (Glubran II®, GEM Srl, Viareggio, Italy). An angiogram performed at the end of the procedure showed exclusion of the aneurysmal sac (Figure 5). CDU and CEUS performed the day after the procedure, showed devascularization of the SAA without any additional ischemic damage to splenic parenchyma. She was discharged the day after with antibiotic coverage without any symptoms related to post-embolization syndrome. A contrast-enhanced CT performed 3 months after the procedure confirmed the complete exclusion of the SAA (Figure 6).

## Discussion

Surgical therapy generally involves the ligature of the splenic artery with splenectomy by laparotomy or laparoscopy; the morbidity and mortality related to the procedure is respectively 9% and 1.3%, rising to 20% in cases of emergency and to 70% during pregnancy [7, 8].

Nowadays, there is general agreement regarding endovascular treatment indications of SAAs [1, 9]. With regard to endovascular approach, embolization can be performed using two techniques: embolization with coils limited to the aneurysmal sac, which maintains continuity in the splenic artery [10, 11] but it is discouraged by some interventional radiologists for the risk of rupture of the aneurysm during manipulation; and endovascular ligature, which requires the positioning of metal coils or plugs [7] in the splenic artery proximal and distal to the SAA until flow is abolished. In the second occurrence, blood flow to the spleen is, at least in part, maintained by collateral vessels [1, 9–11].

Stent graft repair has also been used to treat SAA. This offers the advantage of maintaining patency of the main splenic artery [9, 12]. However, excessive tortuosity of the splenic artery may cause difficulties in the passage of the sheath and stent graft to the SAA location and it may preclude this treatment method [9].

Relative contraindications to interventional radiology treatment include allergy to iodinated contrast media, severe renal impairment, and anatomic issues such as occlusion of the feeding vessels, limited access (for example, occluded femoral/iliac arteries), and challenging local vascular anatomy, which may make transcatheter treatment too difficult to perform [5, 9].

Although splenic ischemia and post-embolization syndrome have been reported in up to 40% of patients undergoing SAA embolization, there have been few clinical sequelae or evidence of hematological changes due to splenic insufficiency [9, 12]. This is explained by the fact that sufficient perfusion of the spleen occurs from collateral flow through the short gastric arteries and from the gastroduodenal artery via the gastroepiploic arteries [9].

When embolizing an aneurysm at the splenic hilum, global concordance exists on the importance of being careful to maintain patency of collateral branches from the short gastric artery and left gastroepiploic artery as well as patency of splenic polar branches originating proximal to the splenic hilum [1, 9]. Some operators [1] confirm patency of these collateral arteries by celiac angiography during balloon occlusion of the splenic artery trunk. Embolization should be planned so as to retain some of the intrasplenic branches because patent collateral branches can prevent total splenic infarction. The intrasplenic branch may originate in, before, or after the aneurysm.

These aneurysms require as much embolization (for example packing with coils) as possible because retrograde blood flow from the intrasplenic branch may result in recurrence of the aneurysm after treatment.

In the case presented, we observed a retrograde blood flow from branches of the left gastric artery not evidenced either by CT or by the first selective and superselective angiography. The most likely hypothesis is that after the first endovascular procedure, the ligature determined an increase of the pressure and its consequent hypertrophy. Reperfusion of the sac was observed as a consequence. In our opinion, a new embolization was mandatory because the risk of rupture of the sac reperfused was higher than originally in relation to higher intrasac pressure.

Our patient represents a rare case of splenic aneurysm reperfused by an efferent derived from her left gastric artery; it was not possible to prevent reperfusion because invasive and noninvasive imaging did not reveal the presence of that vessel. When CT angiography revealed the reperfusion of the sac of the aneurysm without evidence of the real cause (branch of the left gastric artery), time-resolved magnetic resonance angiography was proposed [13] with the advantage of overcoming metal artifacts of coils previously deployed. But, considering the necessity to obtain a complete exclusion of the aneurysm as soon as possible and the hypothesis that the first endovascular ligature was not effective, we decided to perform a new angiography. The evidence of a new vessel afferent to the sac and the correct splenic ligature confirmed the correctness of our choice. The only way to avoid adjunctive ischemic damage of the spleen and of the stomach was to embolize it superselectively.

## Conclusion

In conclusion, when a recurrence of a SAA after treatment is observed, a retrograde blood flow from the intrasplenic branch, not evident at the time of the first treatment, must be considered and investigated.

## Consent

Written informed consent was obtained from the patient for publication of this case report and any accompanying images. A copy of the written consent is available for review by the Editor-in-Chief of this journal.

## Abbreviations

CDU: Color Doppler ultrasound
CEUS: Contrast-enhanced ultrasound
CT: Computed tomography
SAA: Splenic artery aneurysm.
